# Progression of confirmed COVID-19 cases after the implementation of control measures

**DOI:** 10.5935/0103-507X.20200028

**Published:** 2020

**Authors:** Bianca Brandão de Paula Antunes, Igor Tona Peres, Fernanda Araújo Baião, Otavio Tavares Ranzani, Leonardo dos Santos Lourenço Bastos, Amanda de Araújo Batista da Silva, Guilherme Faveret Garcia de Souza, Janaina Figueira Marchesi, Leila Figueiredo Dantas, Soraida Aguilar Vargas, Paula Maçaira, Silvio Hamacher, Fernando Augusto Bozza

**Affiliations:** 1 Departamento de Engenharia Industrial, Pontifícia Universidade Católica - Rio de Janeiro (RJ), Brasil.; 2 Divisão de Pneumologia, Instituto do Coração, Hospital das Clínicas, Faculdade de Medicina, Universidade de São Paulo - São Paulo (SP), Brasil.; 3 Instituto Tecgraf, Pontifícia Universidade Católica - Rio de Janeiro (RJ), Brasil.; 4 Instituto Nacional de Infectologia Evandro Chagas, Fundação Oswaldo Cruz - Rio de Janeiro (RJ), Brasil.

**Keywords:** COVID-19, Coronavirus infections/prevention & control, Pandemics/prevention & control, Infection control/methods, Decision make, Control measure, COVID-19, Infecções por coronavírus/prevenção & controle, Pandemias/prevenção & controle, Controle de infecção/métodos, Tomada de decisão, Medidas de controle

## Abstract

**Objective:**

To analyse the measures adopted by countries that have shown control over the transmission of coronavirus disease 2019 (COVID-19) and how each curve of accumulated cases behaved after the implementation of those measures.

**Methods:**

The methodology adopted for this study comprises three phases: systemizing control measures adopted by different countries, identifying structural breaks in the growth of the number of cases for those countries, and analyzing Brazilian data in particular.

**Results:**

We noted that China (excluding Hubei Province), Hubei Province, and South Korea have been effective in their deceleration of the growth rates of COVID-19 cases. The effectiveness of the measures taken by these countries could be seen after 1 to 2 weeks of their application. In Italy and Spain, control measures at the national level were taken at a late stage of the epidemic, which could have contributed to the high propagation of COVID-19. In Brazil, Rio de Janeiro and São Paulo adopted measures that could be effective in slowing the propagation of the virus. However, we only expect to see their effects on the growth of the curve in the coming days.

**Conclusion:**

Our results may help decisionmakers in countries in relatively early stages of the epidemic, especially Brazil, understand the importance of control measures in decelerating the growth curve of confirmed cases.

## Abstract (pt)

**Objetivo:**

Analisar as medidas adotadas por países que demonstraram controle sobre a transmissão da doença pelo novo coronavírus 2019 (COVID-19) e também como cada curva de casos acumulados se comportou após a implantação dessas medidas.

**Métodos:**

A metodologia adotada para este estudo compreendeu três fases: sistematização das medidas de controle adotadas por diferentes países, identificação dos pontos de inflexão na curva do crescimento do número de casos nesses países e análise específica dos dados brasileiros.

**Resultados:**

Observamos que China (excluindo-se Hubei), Hubei e Coreia do Sul foram eficazes na desaceleração das taxas de crescimento dos casos de COVID-19. A eficácia das medidas tomadas por esses países pode ser observada após 1 ou 2 semanas de sua aplicação. Na Itália e Espanha, foram tomadas medidas de controle em nível nacional em uma fase tardia da epidemia, o que pode ter contribuído para a elevada propagação da COVID-19. No Brasil, Rio de Janeiro e São Paulo adotaram medidas que podem ter sido eficazes na redução da rapidez da propagação do vírus, entretanto, só temos expectativa de ver seus efeitos no crescimento da curva nos próximos dias.

**Conclusão:**

Nossos resultados podem ajudar os responsáveis pela tomada de decisões em países em estágios relativamente precoces da epidemia, especialmente no Brasil, a compreenderem a importância das medidas de controle para desaceleração da curva de crescimento de casos confirmados.

## INTRODUCTION

Coronavirus disease 2019 (COVID-19) was identified in January 2020 in Wuhan, China, and its outbreak was declared a Public Health Emergency of International Concern on January 30, 2020, and a pandemic on March 11, 2020, with more than 118,000 registered cases and 4,000 deaths globally.^(1)^ Since then, the number of confirmed COVID-19 cases has shown exponential growth in different countries, which has resulted in an overload of healthcare systems around the globe. In Brazil, the first case was confirmed on February 25, and the number of contaminated people has increased rapidly, with 1,891 cases confirmed on March 23, 2020.

To mitigate the damages caused to the population, many governments adopted control measures to reduce the levels of transmission, which include the suspension of classes in schools and universities, the prohibition of events and border lockdowns. However, the efficacy of these actions in controlling the progression of the pandemic caused by severe acute respiratory syndrome coronavirus 2 (SARS-CoV-2) is not clear, and specialists debate whether these attempts to flatten the curve of accumulated cases are significant enough to compensate for the financial and social damage incurred.

In this study, we evaluated the progression of accumulated cases of COVID-19 in regions that have implemented control measures to reduce the transmission of the virus. We explored the relation between when and which control measures were adopted and how the curve of accumulated cases behaved after their implementation. This study can assist decisionmakers in countries in relatively early stages of the epidemic, especially Brazil, where control measures have been made sparsely and locally.

## METHODS

We performed an observational study comprised of three phases: systemizing control measures adopted by different countries, identifying structural breaks in the growth of the number of cases for those countries, and specifically analyzing Brazilian data. Our source for the number of cases per day was the World Health Organization, using data provided by John Hopkins University.^(2)^ For Brazil in particular, we used the data provided by the Brazilian Ministry of Health.

The first phase consisted of mapping the information reported by the media concerning the control measures adopted by the countries that had been facing COVID-19 for a longer period of time, that is, long enough to have experienced the effects of the control measures. Data on Hubei Province, the epicenter of the pandemic, were segregated from the data on the other provinces of China and were thus analyzed independently.

Data on these control measures were compared to the time series of confirmed cases (starting on the day that the country reached a total of at least 50 cases) to allow for the analysis of the impact of these measures on the curves of each country. In this phase, we first evaluated the countries that had a historical series that presented a deceleration of the growth rate. Afterwards, we analyzed the numbers of countries that are still in the phase of accelerated disease growth and that took longer to apply measures at a national level. In these cases, the data were also analyzed at a more detailed level (in each region) because control measures were defined and adopted by local authorities.

Then, we identified the points at which the pattern of the time series changed. Therefore, we applied an automatic search of structural changes to each country using the R package *strucchange*.^(3)^ This approach considers a classical linear regression model, as in equation 1.

$$y_{i}=x_{i}^{T}\;\beta +u_{i}$$

where *i* is the index of observations, *y* is the response variable, *x* is the variable that will explain *y*, *β* is the coefficient that connects *y* and *x*, and *u* is the residual. To identify the structural breakpoints, suppose that there are *m* interruption points in *y*, in which the linear regression coefficients vary from one segment to the other. Therefore, there are *m +* 1 segments in which the regression coefficients are constant, and the model can be rewritten as in equation 2.

$$\mathrm{yi}=x_{i}^{T}\;\beta_{j}+u_{i}\;\left(i=i_{j-1}+1\mathrm{,...}i_{j};j=1\mathrm{,...}m+1\right)$$

where *j* denotes the index of the segment. In practice, the breakpoints *i*_*j*_ are rarely obtained in an exogenous form, which is why they need to be estimated. To do so, that is, to define the breakpoints, the residual sum of the squares in Equation (2) is minimized.

Finally, the data from Brazil were analyzed, with a special focus on the top 2 states with the highest number of cases, namely, Rio de Janeiro and São Paulo, with respect to the control measures adopted.

## RESULTS

**Figure 1:**
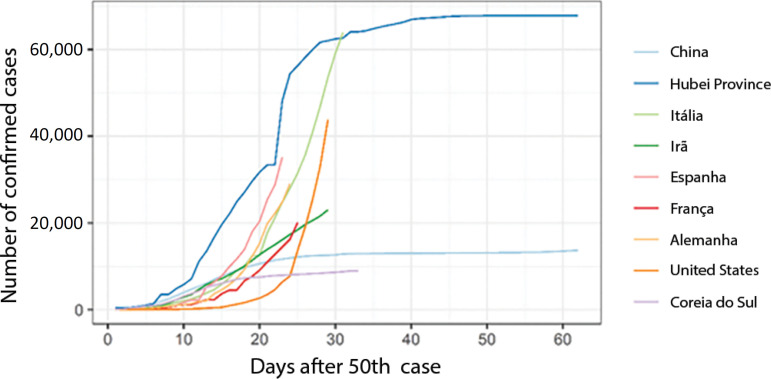
Evolution of the number of confirmed cases, starting on the day that each country reached 50 cases. China’s data do not include those of Hubei Province.

Figure 1 shows the evolution of the number of cases after the 50^th^ case in each country, thus aiding in the selection of the countries to be analyzed. Hubei Province, China (excluding Hubei Province), and South Korea already show a reduction in their growth rates in confirmed cases, even though they adopted different control measures. The other countries apparently do not show signs of stabilization until the 60^th^ day of the epidemic.

Therefore, the curves of the number of cases in Hubei Province, China (excluding Hubei Province) and South Korea, together with their control measures, were separately analyzed. Spain and Italy, which still do not appear to have stabilized their growth rates based on data through March 24, were investigated in greater detail, with regional levels being examined.

### Analysis of effective control measures

#### Hubei Province

**Figure 2:**
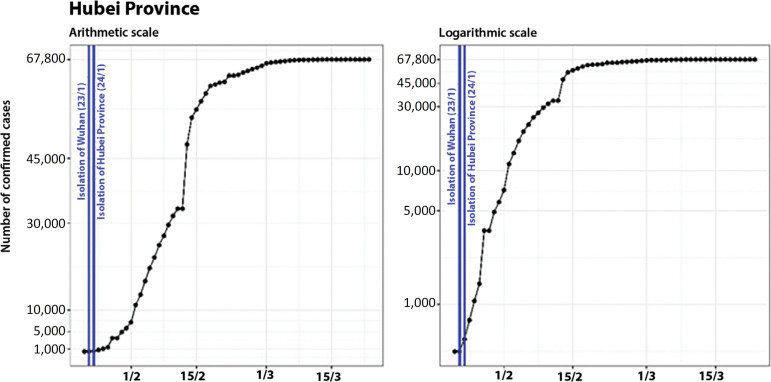
Evolution of the number of confirmed cases in Hubei on arithmetic (left) and logarithmic (right) scales, showing the dates of the control measures.

On January 23, Hubei Province, the epicenter of the pandemic of COVID-19, applied an isolation measure to the city of Wuhan, suspending all public traffic within the city and closing all inbound and outbound transportation.^(4-6)^ On the following day (January 24), 15 other cities in the province were also isolated.^(4,6)^ This measure was taken on the day that Hubei Province reported a total of 444 cases. Figure 2 shows the evolution of the confirmed cases in Hubei Province and the control measures adopted (vertical lines in blue). The left plot shows the number of cases on an arithmetic scale, while the right plot shows them on a logarithmic scale (although the number of cases shown is still the absolute number).

It is possible to see exponential growth in the first phase of the propagation of the disease until the beginning of February, when the curve changed to a linear shape, and posterior attenuation of the curve in the second week of February. We highlight that the numbers on February 11 and 12 were the same, which was probably due to unreported updates.

**Figure 3:**
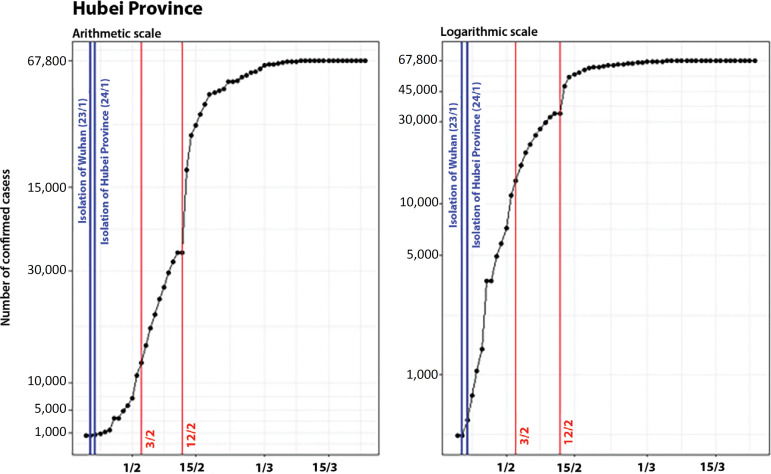
Evolution of the number of confirmed cases in Hubei on arithmetic and logarithmic scales, showing the dates of the control measures and breakpoints.

The breakpoints that indicate the structural changes of the Hubei Province curve are illustrated as vertical red lines in figure 3. They occurred on February 3 (when it changed to linear growth) and February 12 (when it is possible to see the beginning of a period of deceleration of the growth rate, even considering the lack of updates).

On a scale of days, considering t = 0 as the date when the first control measure was adopted, the breakpoints in the curve of confirmed cases occurred on t =11 and t = 20.

### China (excluding Hubei Province)

**Figure 4:**
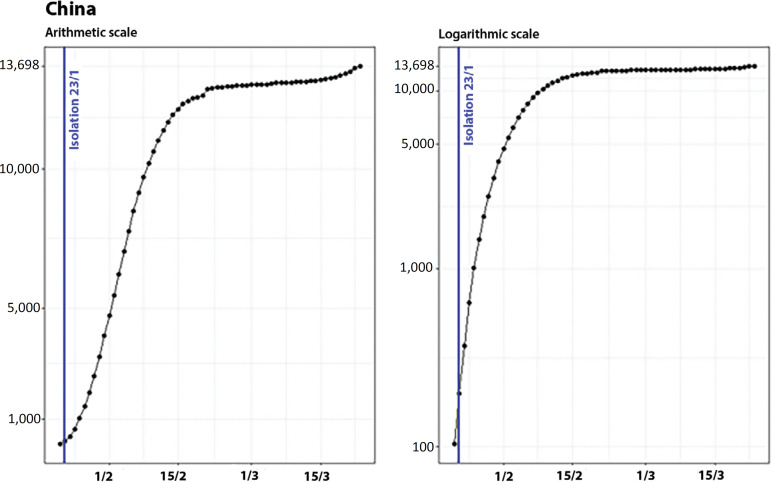
Evolution of the number of confirmed cases in China (excluding Hubei Province) on arithmetic (left) and logarithmic (right) scales, showing the date of the control measure.

At the end of January, China imposed an isolation regime on its inhabitants.^(6,7)^ By that date, it had 199 confirmed cases, and although it was not possible to determine the exact day when the isolation was enforced, we assume that it occurred close to the lockdown of Wuhan’s borders (January 23). Figure 4 shows the evolution of the confirmed cases in China (excluding Hubei Province) and the control measures adopted on arithmetic and logarithmic scales.

Similar to what was perceived in Hubei Province, there was exponential growth in disease propagation in January, while in February, the curve decelerated, and the growth rate attenuated in the second week of the month.

**Figure 5:**
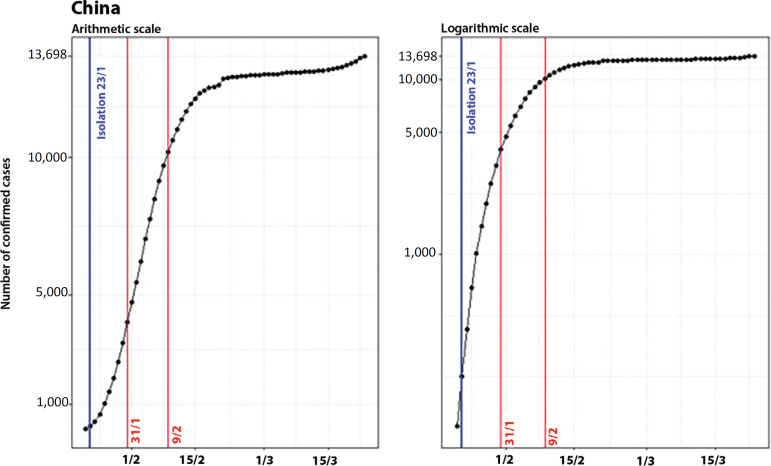
Evolution of the number of confirmed cases in China (excluding Hubei Province) on arithmetic and logarithmic scales, showing the date of the control measure (in blue) and the breakpoints (in red).

The breakpoints in the Chinese curve occurred on January 31 (when the curve concavity changed) and on February 9 (indicating a more expressive decline of the growth rate), as shown in figure 5.

On a scale of days, considering t=0 as the date when the first control measure was adopted, the breakpoints in the curve of confirmed cases occurred on t = 8 and t =17.

### South Korea

**Figure 6:**
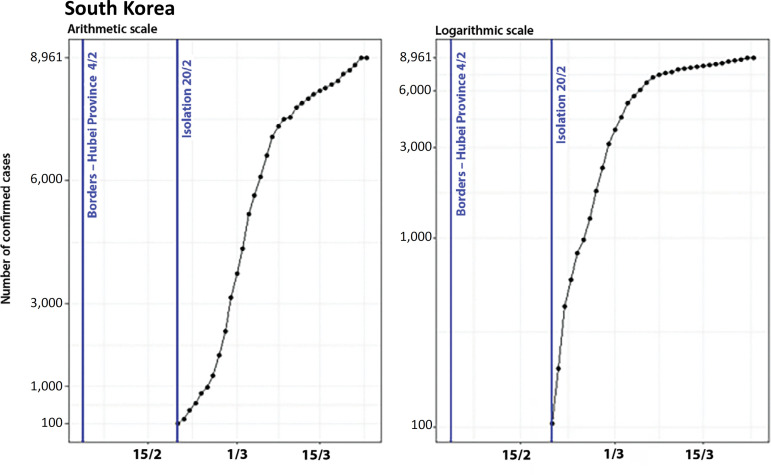
Evolution of the number of confirmed cases in South Korea on arithmetic and logarithmic scales, showing the dates of the control measures.

The prohibition of the entrance of passengers coming from Hubei Province to South Korea occurred on February 4, when the country reported a total of only 16 cases. The recommendation of social isolation^(8)^ was given on February 20, when there were 104 cases. Figure 6 shows the evolution of the number of cases in South Korea and the control measures adopted on both arithmetic and logarithmic scales.

There was a significant period (16 days) in which South Korea had a reduced number of confirmed cases, since its first control measure was adopted (February 4) when it reached more than 50 cases (more precisely, 104 cases on February 20^th^). Since then, we observed exponential growth compatible with the propagation of the virus in the community transmission phase, which lasted until February 28, when a linear increase began. The flattening of the curve started a few days later, in the first week of March.

**Figure 7:**
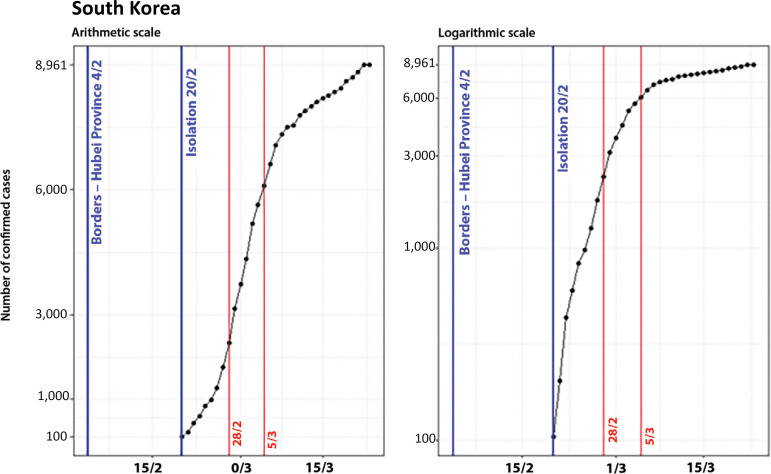
Evolution of the number of confirmed cases in South Korea, on arithmetic and logarithmic scales, showing the dates of the control measures and breakpoints.

The breakpoints of the South Korean curve occurred on February 28 (when the curve concavity changed) and on March 5 (beginning of a new deceleration of the growth rate), as represented in figure 7.

Therefore, on a scale of days, considering t = 0 as the date when the first control measure was adopted, the breakpoints in the curve of confirmed cases occurred on t = 8 and t = 14.

### Analysis of the control measures with no perceived effects (as of March 23, 2020) on the national level

By March 23, 2020, some countries were still experiencing exponential growth in the number of COVID-19 cases, and therefore, there was not enough historical data to analyze the effectiveness of their control measures at the national level, as they were still being implemented or had been recently implemented. This includes Italy and Spain, which we analyze next.

### Italy

**Figure 8:**
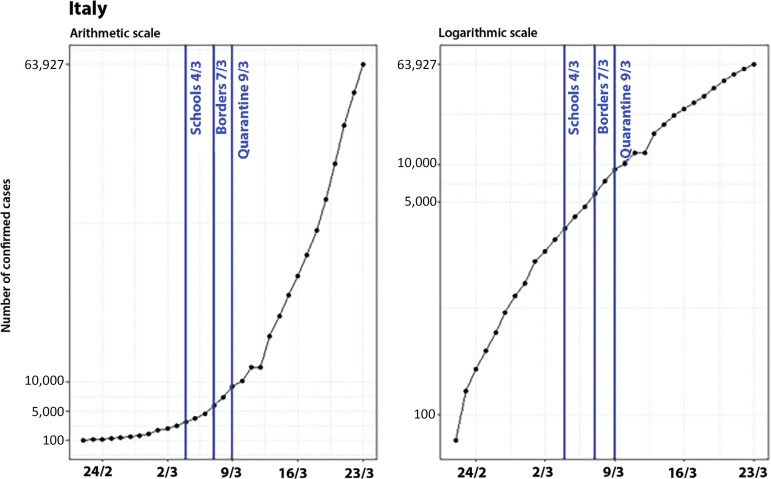
Evolution of the number of confirmed cases in Italy on arithmetic and logarithmic scales, showing the dates of the control measures.

The analysis of what is happening in Italy is more complex than what we have observed in the countries mentioned in the previous section. While in China (excluding Hubei Province), Hubei Province, and South Korea, there were indications that the effectiveness of control measures started 8 to 11 days after their implementation, in Italy, the growing number of cases is still expressive, even after the adoption of a few control measures. Examples of these measures are the suspension of school classes (on March 4, when there were already 3,089 cases), the lockdown of the borders (on March 7; 5,883 cases), and the quarantine implementation (on March 9; 9,172 cases).^(9)^ Therefore, the control measures were only adopted when the country already had an expressive number of confirmed cases (3,089 cases), which might have contributed to reducing the effectiveness of the actions taken (Figure 8).

**Figure 9:**
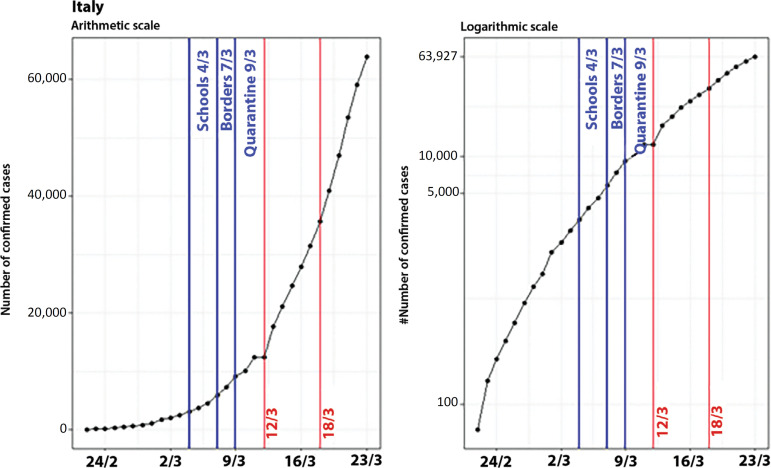
Evolution of the number of confirmed cases in Italy, on arithmetic and logarithmic scales, showing the dates of the control measures and breakpoints.

When we analyze the plot on the arithmetic scale, the measures do not seem to be effective because there are still a substantial number of new cases. Nevertheless, when looking at the growth on the logarithmic scale, we can see a deceleration in the number of new cases, marked by the breakpoints in t = 8 and t = 14, when the suspension of classes is defined as t = 0 (Figure 9).

**Figure 10:**
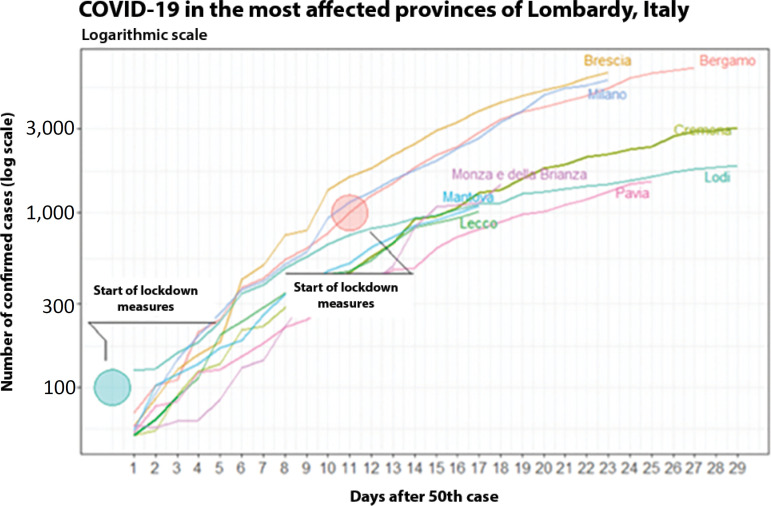
Evolution of the number of confirmed cases in the provinces of Lombardy, Italy, until March 17, 2020 (on a logarithmic scale).

Apparently, these points are not related to school closure, border lockdown, or quarantine. The control measures presented in the graph were actions taken for the entire Italian territory, and it is known that different regions and provinces in Italy adopted measures that precede those at the national level. Therefore, the deceleration of the growth rate might be explained by regional measures. Figure 10 presents the evolution of COVID-19 on a logarithmic scale in the provinces most affected by Lombardy (the epicenter of the Italian epidemic).

The province of Lodi adopted closure measures on February 23, when the number of cases was still less than 100 (considered to be t = 0). However, in Bergamo, these measures only occurred on t = 11 (11 days after the detection of the 50^th^ case in this province).^(10)^ Although Lodi had the highest number of cases at t = 0, we can see a slower evolution of the disease compared to that in other provinces. At approximately t = 7 and t = 14, there was a reduction in the growth rates, similar to those observed in China (excluding Hubei Province), Hubei Province, and South Korea. This comparison reinforces the indications of the effectiveness of the control measures.

### Spain

**Figure 11:**
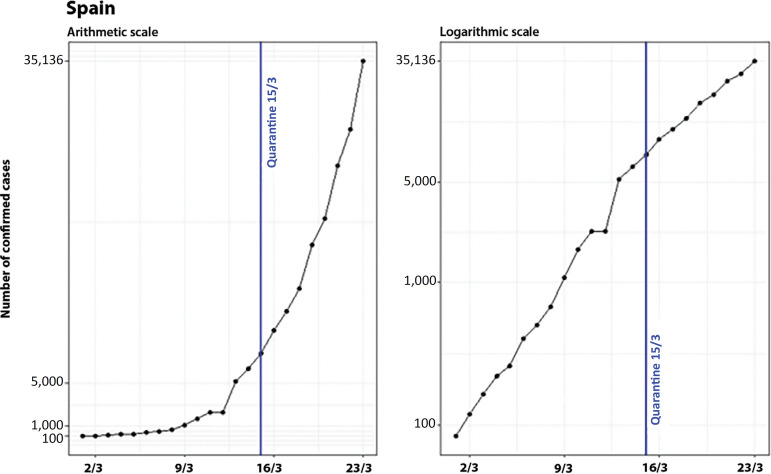
Evolution of the number of confirmed cases in Spain, on arithmetic and logarithmic scales, showing the dates of the national control measure.

Similar to what happened in Italy, the control measures implemented by Spain at a national level were adopted recently (March 15), when the government declared a state of alarm,^(11)^ and, by March 23, it was still not possible to analyze their effects, as shown in figure 11 (also on arithmetic and logarithmic scales).

**Figure 12:**
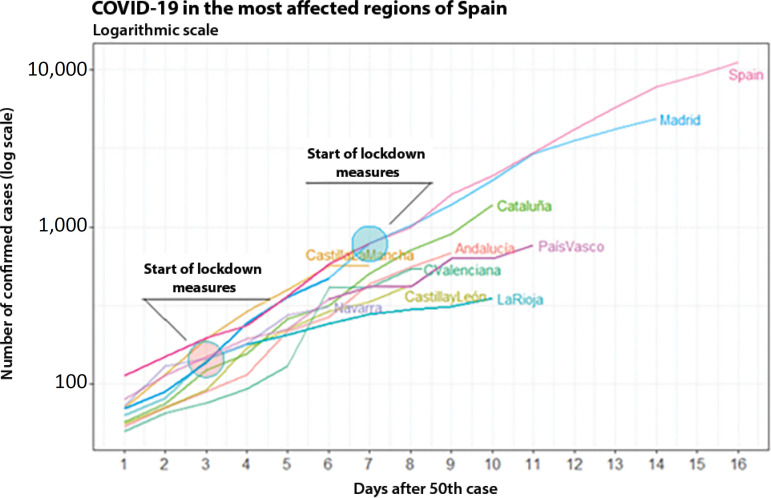
Evolution of the number of confirmed cases in the regions of Spain until March 17, 2020 (on a logarithmic scale).

Therefore, it is important to analyze the progression of COVID-19 in different regions of Spain (Figure 12).

In Spain, three autonomous communities (Madrid, Basque Country, and La Rioja) adopted control measures prior to March 10.^(12)^ Even though the three acts of isolation were taken on the same day, the communities were in different phases of the expansion of the epidemic. While La Rioja and the Basque Country were still at the beginning of development on the third and fourth days after the 50^th^ case, Madrid was already on the seventh day. Figure 12 shows that the evolution of COVID-19 in the regions of La Rioja and the Basque Country was slower than in other areas of Spain. This difference reinforces the indications of the effectiveness of the control measures and the importance of not only timing but also the number of diagnosed cases, which indirectly measure the transmission occurring in the community.

### Brazil

**Figure 13:**
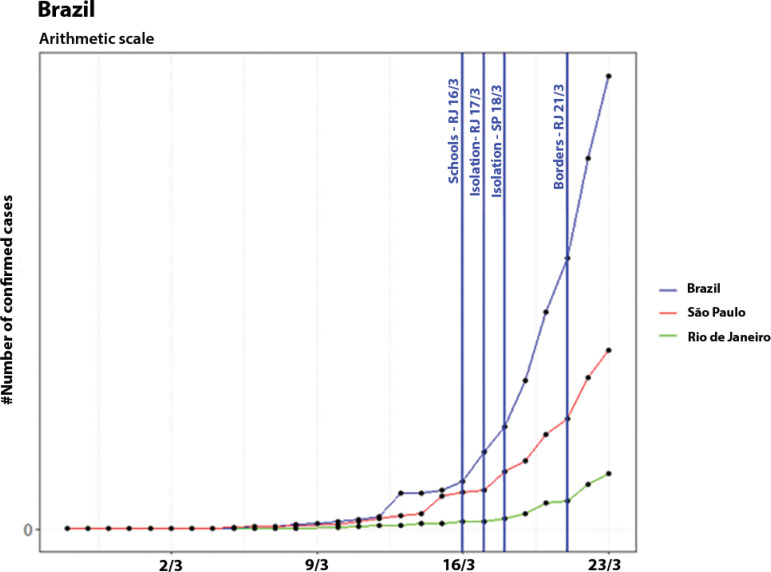
Evolution of the number of confirmed cases in Brazil and in the states of Rio de Janeiro and São Paulo, on an arithmetic scale, showing the dates of the control measures.

In Brazil, epidemic progression is still in a phase of exponential growth, and there are not enough historical data to analyze control measures at the national level. However, following the example of other countries, some states have adopted control measures to slow down the growth of the curve. Figure 13 illustrates the total number of confirmed cases in Brazil and in the two states with the highest number of cases: Rio de Janeiro and São Paulo. The control measures adopted by these states are also illustrated.

The state of Rio de Janeiro suspended classes on March 16^(13)^ and adopted social isolation on March 17.^(14)^ The state of São Paulo adopted social isolation on March 18, and even though it had almost five times the number of confirmed cases of Rio de Janeiro (on March 17), it took longer to suspend classes (March 23).^(15)^ Moreover, the state of Rio de Janeiro implemented a border lockdown on March 21. Regarding social isolation measures, both states recommended the closure of shopping centers and gyms and the suspension of all events.

## DISCUSSION

The data analysis results indicate that high-impact control measures (such as social isolation and quarantine), which were adopted in China (excluding Hubei Province), Hubei Province, and South Korea, have been effective in the deceleration of the growth rates of COVID-19 cases. The evolution of the epidemic in these countries indicates that the effectiveness of these measures begins after 1 to 2 weeks of their application. In the analyzed countries, the first deceleration in the growth curve occurred after 8 to 11 days, while the second deceleration occurred 14 to 20 days after the first control measure was taken.

These conclusions are in line with the work of Anderson et al.^(16)^ They stated that quarantine, social distancing, and the isolation of infected populations were responsible for containing the epidemic in China and that the result of these actions should inspire countries in which the disease was beginning to spread. They also noted that individual behavior can be crucial to control the spread of COVID-19, in which personal attitudes, such as self-isolation and social distancing, might be even more relevant than government impositions, especially in Western democracies.

The analysis of the control measures adopted by South Korea presents some peculiarities. First, the actions were taken at the beginning of the pandemic, when the number of confirmed cases was very low. The slow growth could indicate that the initial control measure adopted in the beginning of the epidemic might have postponed the propagation of the disease. Another important factor to consider is that South Korea used massive testing as an additional strategy to identify more cases in the community, which is a measure considered to be effective but not always possible on a large scale. Additionally, it is plausible that cultural aspects of the South Korean population may have contributed to the high effectiveness of the recommendation of social isolation.

In Italy and Spain, it took longer for control measures to be adopted at the national level, and by the time they were adopted, the epidemic was already at an advanced stage, which could have reduced the effectiveness of these measures. The regions where local measures were taken in the early days of the epidemic (Lodi in Italy; La Rioja and the Basque Country in Spain) presented indications of reduction in the propagation of the disease.

Regarding the evolution of COVID-19 in Brazil, the states with the highest number of cases (Rio de Janeiro and São Paulo) adopted measures that could be effective in slowing the propagation of the virus. However, we only expect to see their effects on the growth of the curve in the coming days. Given that the measures in Rio de Janeiro were taken earlier than those taken in São Paulo (especially when we compare the number of cases that each state had when they were adopted), we believe that the containment in Rio will be more effective. We can also note that the country still has not adopted a national control measure, which may hinder the retention of the disease in Brazil.

We should take into consideration that the analyzed data concern confirmed cases. It is estimated that symptoms of COVID-19 infection might take up to 14 days to show. In studies performed in China, the median time period has been between 4 and 5 days. Therefore, the analysis of confirmed cases and decision-making are performed at least 4 days later. In addition, the effects seen on the growth rates might have been influenced by other factors not listed here, thus requiring further investigation.

It is important that Brazil benefits from having to experiment with the beginning of the virus propagation after other countries have already done so. Therefore, the analyses made in this study can help in the decision-making process and function as evidence of the effects of the control measures. In addition, the examples of other countries and regions were also analyzed regarding different types of measures, varying in their coverage level (local, regional, or national), dosage (moderate or radical impact), readiness (prevention or mitigation), level of authority imposition (recommendations or prohibitions) and cultural aspects.

## CONCLUSION

In this paper, we presented and analyzed data and information from the World Health Organization on the control measures adopted by other countries, as well as the changes in the growth rates observed in the number of COVID-19 cases. In addition, we commented on the recent control measures adopted in Brazil, specifically in the states of Rio de Janeiro and São Paulo, compared to what was observed in other countries. Our analysis may help decisionmakers in countries in relatively early stages of the epidemic, especially Brazil, understand the importance of control measures in slowing down the growth curve of confirmed cases.
